# Expanding Spectrum of *FIG4* ‐Related Neurological Disorders of Lysosomal Homeostasis: Case Report and Overview of the Potential Genotype–Phenotype Correlations

**DOI:** 10.1111/cge.70185

**Published:** 2026-05-22

**Authors:** Pankaj Prasun, Matthew Rasberry

**Affiliations:** ^1^ Department of Pediatrics, Division of Genetics and Metabolism University of Wisconsin‐Madison Madison Wisconsin USA

**Keywords:** amyotrophic lateral sclerosis, Charcot–Marie–Tooth disease, *FIG4*, hypomyelination, juvenile ossifying fibroma

## Abstract

Biallelic loss‐of‐function variants in *FIG4* are associated with Charcot–Marie–Tooth disease type 4J, a progressive peripheral sensorimotor demyelinating polyneuropathy. Biallelic null *FIG4* variants cause Yunis‐Varon syndrome, a severe neurological disorder characterized by global developmental delay, hypotonia, brain malformations, skeletal defects, dysmorphic facial features, and juvenile lethality. In the past few years, many individuals with combined central and peripheral nervous system disease associated with biallelic *FIG4* variants have been described. In addition, certain heterozygous *FIG4* variants are associated with amyotrophic lateral sclerosis. We describe an individual with global developmental delay, hypotonia, cerebral hypomyelination, peripheral hypomyelinating polyneuropathy, frequent fractures, and juvenile ossifying fibroma. The spectrum of clinical presentation of *FIG4*‐related disorders is increasingly being recognized. Our observations expand the phenotypic spectrum of *FIG4*‐related neurological disorders. In addition, we provide an overview of the potential genotype–phenotype correlations of this expanding group of disorders of lysosomal homeostasis.

## Graphical abstract

*FIG4* is essential for lysosomal homeostasis. *FIG4*‐related disorders present as a continuous spectrum from the juvenile lethality in Yunis‐Varon syndrome to an increased risk of amyotrophic lateral sclerosis (ALS) in adult life. *FIG4*‐related disorders comprise a novel group of disorders of lysosomal homeostasis and can be classified into severe, intermediate, and mild presentations like many other lysosomal disorders.

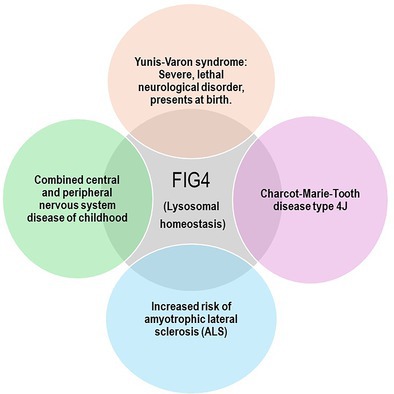

## 1 Background

*FIG4* (FIG4 phosphoinositide 5‐phosphatase) (OMIM 609390) was first associated with Charcot–Marie–Tooth disease type 4J (CMT4J; OMIM 611228) [1]. CMT4J presents with progressive demyelinating sensorimotor peripheral neuropathy. The age of presentation varies from early childhood to adulthood. It is caused by biallelic loss‐of‐function *FIG4* variants. Often, there is compound heterozygosity for a null and the p. Ile41Thr missense variant [2]. Biallelic homozygous or compound heterozygous null *FIG4* variants cause Yunis–Varon syndrome (YVS; OMIM 216340), a severe neurological disorder characterized by brain malformations, hypotonia, skeletal defects, facial dysmorphism, and juvenile lethality [3]. Apart from these disorders of peripheral (CMT4J) and central (YVS) nervous systems, *FIG4* is associated with other neurological conditions. Development of parkinsonism in several CMT4J individuals has been described [4, 5, 6]. The combination of peripheral neuropathy and central nervous system involvement (cognitive deficits, ataxia, parkinsonism, seizure) in some individuals suggested a phenotypic continuum between YVS and CMT4J [5]. A few individuals with novel compound heterozygous variants presented with developmental delays, hypotonia, leukoencephalopathy, and peripheral neuropathy [7]. Novel homozygous p. Tyr169Ser variants were found in individuals with cognitive deficits, peripheral neuropathy, dystonia, hypotonia, and swallowing difficulties. Brain MRI showed cerebellar atrophy and bilateral T2 hyperintense medullary changes suggesting a distinct clinicoradiological phenotype [8]. Two Chinese siblings with novel compound heterozygous variants presented with global developmental delay, progressive muscle weakness, and swallowing difficulty [9]. Neuroimaging showed thinning of corpus callosum, but no demyelination/hypomyelination or medullary changes. Progression to supranuclear palsy has been described in an individual with CMT4J who was compound heterozygous for the p. Ile41Thr variant and a novel nonsense variant (p. Trp246*) [10]. Surprisingly, a previously healthy 44‐year‐old individual who harbored homozygous p. Ile41Thr variant presented with rapidly progressive dystonia and parkinsonism after head trauma [11]. In a consanguineous Moroccan family, homozygous p. Asp783Val missense variant was associated with bilateral temporooccipital polymicrogyria with seizures and psychiatric manifestations [12]. Moreover, certain heterozygous variants in *FIG4* are associated with amyotrophic lateral sclerosis type 11 (ALS11; OMIM 612577), a progressive fatal neurological disorder characterized by combined upper and lower motor neuron features [13, 14].

We describe an individual with global developmental delay, muscle weakness and atrophy, hypotonia, areflexia, peripheral and central hypomyelination, and swallowing dysfunction who developed juvenile ossifying fibroma (JOF). Our observations endorse the phenotypic continuum of *FIG4*‐related disorders and broaden its phenotypic spectrum. In addition, we provide an overview of the potential genotype–phenotype correlations for *FIG4*‐related neurological disorders.

## 2 Methods

This retrospective observational study does not require ethics committee approval at this institution. The legal guardian of the proband provided written consent for publication.

## 3 Case Presentation

This individual, an 18‐year‐old male, was born to a 33‐year‐old G4P2 mother via C‐section at 38 weeks of gestation. Antenatal, birth, and neonatal periods were unremarkable except for low amniotic fluid detected at 26 weeks gestation. At 2 months of age, he contracted a respiratory illness requiring mechanical ventilation. After recovery, profound hypotonia was noted. Brain MRI showed prominent sylvian fissures and small frontal lobes. High triglycerides in blood and very high glycerol in urine suggested glycerol kinase (GK) deficiency. Complex GK deficiency due to contiguous gene deletion at chromosome Xp21 encompassing the genes for GK deficiency and Duchene muscular dystrophy (DMD) was suspected due to severe hypotonia. However, fluorescence in situ hybridization (FISH) analysis for Xp21 deletion was normal, ruling out this possibility. Early in infancy, visual inattention and nystagmus were noted. He continued to have global developmental delay, profound hypotonia, and areflexia, prompting a detailed neurogenetic evaluation at 13 months of age. Prominent metopic suture, bilateral epicanthal folds, micrognathia, high arched palate, and myopathic facies were noted on genetic evaluation. Family history was significant for rapidly progressing ALS in maternal great uncle. Methylation studies for Angelman/Prader Willi syndrome, carbohydrate deficient transferrin assay, and lysosomal enzyme assays were normal. Brain MRI demonstrated severe hypomyelination. Electromyography/nerve conduction velocity showed severe axonal sensorimotor demyelinating polyneuropathy. Sural nerve biopsy demonstrated markedly decreased myelination and no “onion bulb” formation suggestive of congenital hypomyelinating neuropathy (CHN). However, CHN gene panel was normal. Muscle biopsy was unremarkable on light microscopy. Electron microscopy showed rare enlarged mitochondria with abnormal cristae. Mitochondrial assays on fresh frozen muscle biopsy specimen showed decreased respiratory chain complex II and II–III activities. Muscle coenzyme Q10 level was reduced. Based on these findings, a provisional diagnosis of mitochondrial myopathy was made. However, mitochondrial genome sequencing and a panel of nuclear genes associated with mitochondrial diseases were normal. Chromosome microarray showed intragenic deletion involving exon 19 of the *GK* gene associated with GK deficiency. Continuous feed containing high carbohydrate and low fat was started due to this diagnosis. Severe feed intolerance led to fundoplication and gastrostomy tube insertion followed by conversion to gastrojejunostomy tube.

**FIGURE 1:**
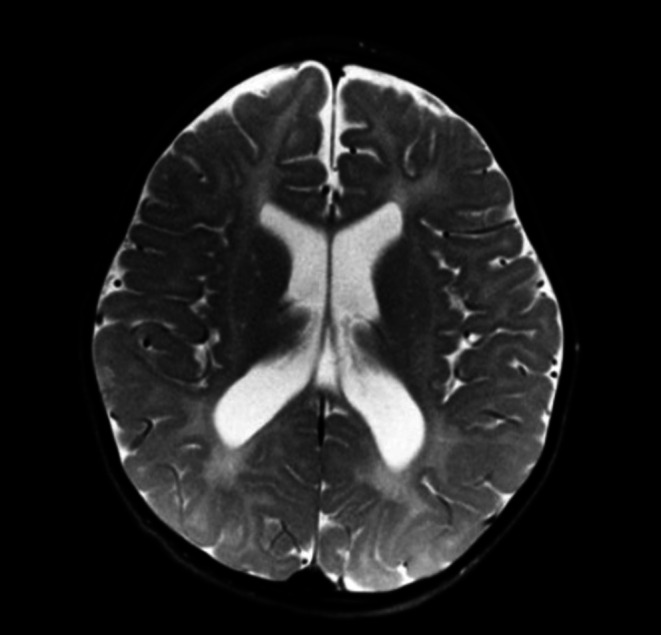
Brain MRI at five and half years of age showing significant hypomyelination in T2‐weighted axial image.

The patient continued to have profound developmental delays. He developed sensorineural hearing loss and became legally blind by 3 years of age. He had recurrent respiratory and ear infections. Some of the infection episodes were associated with hypoglycemia. At 5.5 years of age, he developed choreoathetoid movements. Brain MRI showed severe hypomyelination (Figure 1), electroencephalogram (EEG) was normal. Eye examination under anesthesia showed retinitis pigmentosa and bilateral optic atrophy. At 6 years of age, he developed bilateral jaw swelling suspicious for fibrous dysplasia on CT maxillofacial region. Biopsy of the growth was diagnostic of JOF. He underwent debulking surgery, but recurrence after 6 months required another debulking procedure. However, the growth recurred again. Treatment with Zoledronic acid infusions was started without much improvement. Hence, it was switched to Denosumab injections, which led to stabilization. Frequent fractures while on Denosumab led to its discontinuation after about 6 years of therapy. Denosumab withdrawal led to hypercalcemia precipitating acute pancreatitis and progression to chronic pancreatitis. Recently, the patient has developed staring spells, which is gradually worsening in frequency and duration. The recent staring spells are associated with abnormal breathing, temperature instability, asymmetric pupillary dilation, and excessive sweating. The patient has also started to have generalized tonic–clonic seizures. The most recent continuous 44‐h EEG showed diffuse slowing in delta/theta range indicative of nonspecific encephalopathy. In addition, there were infrequent interictal spike wave discharges with left predominance.

Exome sequencing was obtained at the age of 8 years. It showed compound heterozygous pathogenic variations in *FIG4*. A splice donor variant, c.2459 + 1G > A (IVS21 + 1G > A) was paternally inherited, while a null variant c.831_838delTAAATTTG (p. Lys278Trp fs*6) was maternally inherited. The intragenic deletion involving exon 19 of *GK* was redemonstrated. It is a *de novo* finding. The parents are asymptomatic.

## 4 Discussion

FIG4 is part of a protein complex that regulates the level of phosphatidylinositol (3,5)‐bisphosphate (PI (3,5) P2) on the lysosomal membrane [15]. PI (3,5) P2 plays an essential role in maintaining lysosomal homeostasis by regulating several lysosomal membrane ion channels and transporters [16]. FIG4 deficiency is associated with enlarged, vacuolated lysosomes in neurons and muscle [3]. FIG4 deficiency should be viewed as a disorder of lysosomal homeostasis.

FIG4 deficiency was previously associated with distinct peripheral (CMT4J) and central (YVS) nervous system diseases. The broad spectrum of FIG4 deficiency is increasingly being recognized due to the widespread use of genomic technologies in clinical diagnostics. It can be divided into severe, intermediate, and mild presentations. The severity of clinical presentation is most likely determined by the residual FIG4 activity determined by the genotype. Variable/unique presentation could be related to a particular genotype. Table 1 summarizes the potential genotype–phenotype correlations of *FIG4*‐related neurological disorders.

**TABLE 1:** Overview of the potential genotype–phenotype correlations in *FIG4*‐related neurological disorders of lysosomal homeostasis.

Mode of inheritance	Severity	Clinical features	Prognosis	Genotype–phenotype correlation	References
Autosomal recessive	Severe	*Yunis–Varon syndrome*: Brain malformations, hypotonia, skeletal defects (cleidocranial dysplasia, absence of halluces/thumbs, distal aphalangia, gracile bones, frequent fractures), facial dysmorphism (sparse scalp and eyebrow hairs, protruding eyes, high arched palate, micrognathia, thin upper lip, low set ears), retinopathy. Neuropathology shows extensive neuronal loss involving corpus callosum, basal ganglia, frontal lobes, and cerebellar vermis. Enlarged vacuoles seen in neurons, muscle, cartilage, and other tissues.	Individuals with this condition present at birth and most die in the infancy or early childhood.	Homozygosity or compound heterozygosity for null *FIG4* variants.	Campeau et al., 2013.
	Intermediate	Global developmental delay, hypotonia, cerebral hypomyelination, cerebellar atrophy, medullary swelling, maculopathy/retinopathy, areflexia, peripheral hypomyelinating polyneuropathy.	Disease onset in the infancy to early childhood. Compatible with survival into childhood or early adulthood.	Compound heterozygosity for a null variant and a partially functional variant (splice site variant, variant at an evolutionary conserved site, or an enzymatically active site). Homozygosity for a splice site variant or a variant at an evolutionary conserved site such as the p. Tyr169Ser variant.	Lenk et al., 2019. Wright et al., 2020. Yu et al., 2022.
	Mild	*CMT4J*: Progressive peripheral demyelinating sensorimotor polyneuropathy. Subtle dysmorphic facial features may be present. Some of the individuals may have mild cognitive deficit at the onset or may develop other neurological complications such as parkinsonism, ataxia, supranuclear palsy or seizures later in life. Rapid onset of dystonia‐parkinsonism after head injury in a previously healthy adult has also been described.	Disease onset from childhood to adulthood. Compatible with survival into adulthood.	Compound heterozygosity for a null/partially functional/splice site or other intronic variant and the p. Ile41Thr variant. Homozygosity for the p. Ile41Thr variant or a partially functional variant.	Georgiades et al., 2025. Lauerova et al., 2025. Orengo et al., 2018. Retailleau et al., 2025. Sadjadi et al., 2024. Zimmermann et al., 2020.
	Unique presentation	Cortical malformations (temporo‐occipital polymicrogyria), epilepsy, psychiatric manifestations.	Clinical presentation from birth to adulthood. Outcome depends on compliance with the antiepileptic medications. Sudden death occurred in untreated patients.	Homozygosity for the p. Asp783Val missense variant.	Baulac et al., 2014
Autosomal dominant	Severe	*Amyotrophic lateral sclerosis (ALS) type 11*: Progressive degeneration of upper and lower motor neurons resulting in paralysis, respiratory failure, and death.	Disease onset in middle to old age. Rapidly progressive and fatal.	Heterozygous deleterious variant as risk allele. *FIG4* deleterious heterozygous variants may account for up to 3% of ALS patients.	Chow et al., 2008. Osmanovic et al., 2017.

The patient had onset of illness in infancy with hypotonia, muscle weakness, vision impairment, nystagmus, and developmental delays. He has both peripheral and central hypomyelination. He fits into the intermediate presentation. He has compound heterozygous c.831_838delTAAATTTG (p. Lys278Trp fs*6) and c.2459 + 1G > A (IVS21 + 1G > A) *FIG4* variants. c.831_838delTAAATTTG variant causes an 8‐bp frameshift deletion known to be associated with YVS [3]. The c.2459 + 1G > A variant affects splicing, a known partial loss of function variant [7]. This variant has been reported with intermediate presentation when present in homozygous or compound heterozygous form with a null variant [7]. Initially, the patient was diagnosed with CMT4J, and his developmental delay was attributed to the hypoglycemic episodes secondary to GK deficiency [17]. Retrospectively, it is apparent that his global developmental delay is secondary to FIG4 deficiency and not the GK deficiency. Several case series and reports of FIG4 deficiency describe developmental delay/intellectual disability as part of the clinical presentation [5, 6, 7, 8, 9]. Intellectual impairment/cognitive deficits have also been documented in many individuals with CMT4J [2].

GK deficiency is classified into complex and isolated types. Complex GK deficiency is due to contiguous gene deletion at chromosome Xp21.2 involving the genes *GK*, *DMD*, and *NR0B1*. It is associated with muscular dystrophy, adrenal failure, and developmental delays in addition to the GK deficiency. This was ruled out by FISH analysis. The patient has a *de novo* intragenic deletion involving exon 19 of *GK* associated with isolated GK deficiency [18]. Isolated GK deficiency is not associated with hypotonia or developmental delays. It is divided into juvenile and adult‐onset types. The juvenile‐onset type may present with hypoglycemia precipitated by metabolic stressors such as infections during childhood. Symptoms usually disappear with advancing age. The adult‐onset GK deficiency is usually asymptomatic and detected incidentally by pseudo‐hypertriglyceridemia on routine tests. The hypoglycemia episodes during childhood in this patient were likely due to GK deficiency. He most likely has the juvenile‐onset type which has improved consistent with the natural history of this condition.

Skeletal manifestations are common in YVS, the most severe form of FIG4 deficiency. Apart from skeletal defects, YVS patients demonstrate osteopenia and frequent fractures [3, 19]. Vacuoles compatible with enlarged lysosomes were also seen in cartilage in addition to neurons and muscle in YVS [3]. Vacuolization was demonstrated in osteoblasts of FIG4‐null mice [3]. Osteoblast dysfunction leading to reduced bone formation is the most likely etiology of osteopenia and fractures in YVS [19]. The patient had multiple fractures likely due to osteoblast dysfunction. He has JOF, which is an aggressive form of ossifying fibroma characterized by bone resorption and a tendency to recur. There is increased osteoclastic activity in JOF due to increased receptor activator of nuclear factor‐κB ligand (RANKL), which binds to the receptor activator of nuclear factor‐κB (RANK) on osteoclast precursors, promoting its differentiation and activation [20]. The patient showed a good response to Denosumab, which is an anti‐RANKL antibody and thus inhibits osteoclastic maturation and activity. JOF has not been reported in *FIG4*‐related disorders. However, it could be related to FIG4 deficiency. Macrophages and fibroblasts are the main sources of RANKL, and its level is negatively correlated with the autophagy in fibroblasts [21]. Hence, impaired autophagy due to impaired lysosomal function in FIG4 deficiency will increase RANKL level and could be the key pathogenetic mechanism of JOF formation in FIG4 deficiency.

This individual's maternal great uncle passed away from rapidly progressive ALS. His genetic status is unknown. However, the c.831_838delTAAATTTG (p. Lys278Trp fs*6) variant is maternally inherited. It is possible that this variant increases ALS risk. This variant has not been reported with increased ALS risk, but similar frameshift truncating variants have been implicated [13, 14]. Lysosomal dysfunction is implicated in the pathogenesis of ALS [22]. A better understanding of FIG4‐related neurological disorders may open novel therapeutic avenues for this devastating neuromuscular disorder of adult‐onset.

## 5 Conclusion

*FIG4* deficiency comprises a novel group of neuromuscular disorders of lysosomal homeostasis which presents as continuous spectrum from congenital lethal to milder adult‐onset disorders. JOF could be a hitherto unrecognized feature of this disorder. Skeletal manifestations of *FIG4*‐related disorder highlight the role of lysosome in bone physiology and pathology.

## Author Contributions

**Pankaj Prasun:** conceptualization, investigation, writing – original draft, writing – review and editing, supervision. **Matthew Rasberry:** investigation, writing – review and editing.

## Funding

The authors have nothing to report.

## Ethics Statement

This report is a retrospective clinical observation. Ethical approval was not required for this study in accordance with local/national guidelines.

## Consent

Written informed consent was obtained from the parent/guardian for publication of the details of their child's medical case and any accompanying images.

## Conflicts of Interest

The authors declare no conflicts of interest.

## Data Availability

Data sharing not applicable to this article as no datasets were generated or analysed during the current study.
